# The future of myelodysplastic syndrome—patient priorities and outcomes that matter

**DOI:** 10.3389/fmed.2023.1267139

**Published:** 2023-12-18

**Authors:** Matthew Poynton, Catriona Gilmour-Hamilton, Isabella Dale-Harris, Evelyn Clarke, Simon Stanworth, Mike Murphy, Noémi Roy

**Affiliations:** ^1^Churchill Hospital, Oxford University Hospitals NHS Trust, Oxford, United Kingdom; ^2^NHS Blood and Transplant, Radcliffe Department of Medicine, Medical Sciences Division, University of Oxford, Oxford, United Kingdom

**Keywords:** myelodysplastic syndrome (MDS), transfusion, patient focus groups, qualitative research, patient focused care, quality of life

## Abstract

**Background:**

Without a definitive curative option available to many patients, learning to live with myelodysplastic syndrome (MDS) and manage symptoms effectively becomes a priority in their care. Anaemia is an almost universal feature of MDS. Individuals suffer differently and better individualisation of care is needed. Most MDS patient information offers scant appreciation for disease heterogeneity, variable response to treatment and each patient’s likely trajectory.

**Methods:**

We undertook a two-part, online workshop to discuss what matters most to people living with MDS. Patients generated questions about their condition which they felt should be addressed by research or change how their care is delivered. Patients voted on the importance of each topic, creating a “prioritised” list of issues.

**Results:**

Fourteen participants of varying age and experience took part raising 56 unique questions under the themes of: prognosis; end of life; treatment; supportive care; medical staff training; diagnosis and communication. These reflect the symptoms of MDS, improving quality of life (QoL) and communication.

**Discussion:**

Although haemoglobin (Hb) levels have correlation to QoL, it is widely reported that other factors are important in determining QoL and need for transfusions varies despite stable Hb levels. We showed that Hb level and the need for transfusions is not comparable between different patients and even non-comparable over time meaning that the maximal benefit and timing of transfusions cannot be determined from Hb alone. This workshop highlighted patient dissatisfaction with the “numbers-led” approach and the need for an alternative method to determine when to transfuse.

## Introduction

Myelodysplastic syndrome (MDS) is a rare disease but has an increasing prevalence in the elderly population with an average age of 71 (1). With no definitive curative option available to many patients, learning to live with MDS, manage symptoms effectively and improve quality of life (QoL) are priorities in their care. Symptoms from anaemia are an almost universal feature of MDS but other cytopenias are common and may cause symptoms of bleeding and infection. Individual patients’ experience of these symptoms is variable and there is limited appreciation of the heterogeneity of the disease or the trajectory of patients’ journeys with MDS in current written patient information.

This article summarises the findings of a novel, patient-centred two-part online workshop to determine what matters most to people living with MDS and the feedback provided during discussions in patient support programmes. The aim of the workshops was to inform and align lived experience views with research prioritisation in MDS.

## Methods

People with MDS from local and national support groups in the UK were invited to attend. We had hoped to attract 20 participants, as this is a number that remains manageable in a Zoom environment. By the time of the first workshop, we had 14 confirmed participants with a median age of 72. The people who took part were of varying age and experience. There were no inclusion criteria other than having a diagnosis of MDS. 13/14 participants had low or intermediate-1 IPSS risk scores.

We conducted a qualitative two-part interactive online workshop to adapt the face-to-face approach to the online environment. We used an online platform with the assistance of a skilled technical facilitator, to replicate some of the key characteristics of a face-to-face discussion: the synergy of a room full of people, the physical creation of a wall of ideas, and the jostle to pin your choices to questions in voting for the top priorities. To manage “Zoom fatigue” and acknowledging the likely challenge of fatigue in the older age group of the participants, the workshop was divided into two sessions each of two and a half hours duration.

Patients were invited to generate questions about their condition which they felt should be addressed by research or promote change in how their care is delivered. This took the form of an open, non-directed discussion with the aim not to draw on any pre-existing bias. Following small group discussions, these patient-generated questions were then grouped into themes and reframed to create non-overlapping topics to be addressed. This was summarised in advance of the second session and questions to represent issues raised within the identified themes were formed. These questions were shared with participants in advance of the 2nd workshop, and individuals were invited the team with any comments or additions should the questions not represent what they had wanted to contribute.

The second meeting was dedicated to discussion, further grouping of the questions under umbrella headings, and then voting. Using a model inspired by the James Lind Alliance Priority Setting Partnerships (2), patients then voted on the importance of each topic, creating a final “prioritised” list of issues. For the final half hour, the group were able to discuss the results of the workshops with four practicing clinicians and two nurse specialists.

## Results

Fourteen participants of varying age and experience took part across two sessions, with issues raised within the identified themes. Fifty-six unique questions and comments were elicited through patient-directed group discussion under the themes of: prognosis; end of life; treatment; supportive care; medical staff training; diagnosis; communication; organisations and resource sharing.

**Figure 1:**
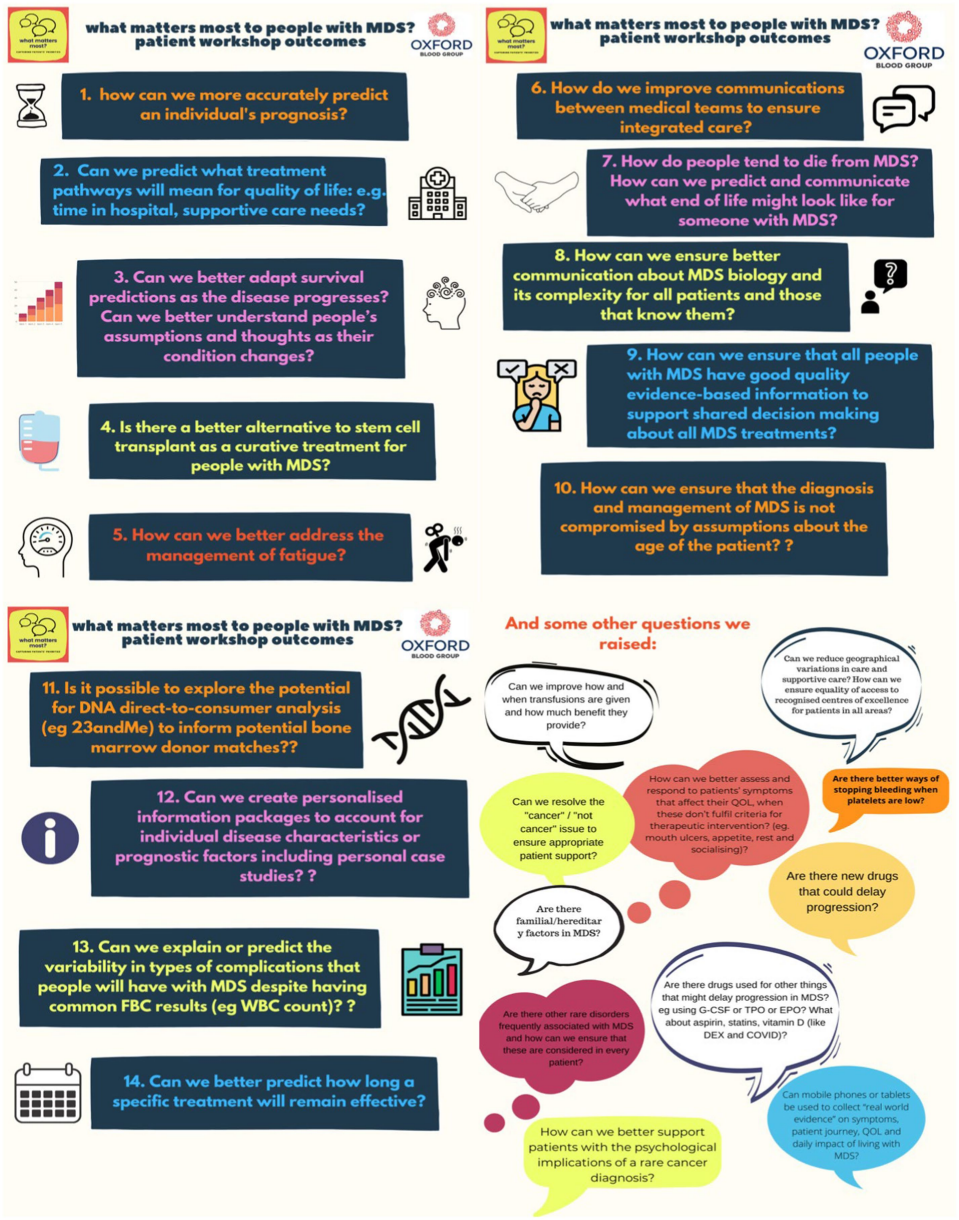
Fourteen key patient workshop outcomes and other issues raised.

Using the voting system, patients living with MDS prioritised 14 key outcomes (shown in Figure 1). Their first thoughts indicate that patients are most concerned with how to live well with MDS. These reflect the symptoms of MDS and how to improve QoL, communication and access to healthcare professionals. Much discussion also focused on supportive therapies and the rationale for using treatments such as blood transfusions. We discussed the frustrations of being treated according to a blood test result rather than listening to patients and treating them according to their symptoms. Although haemoglobin (Hb) levels have been found to be correlated to quality of life (3), it is widely reported that other factors are important in determining QoL and need for transfusions varies despite stable Hb levels (4). Indeed, a key finding from patient discussions showed that Hb level and the need for transfusions is variable over time even in individual patients and not comparable between different patients suggesting that the optimal benefit and timing of transfusions cannot be determined from Hb level alone.

Finally, both the group and clinicians found the shared last session advantageous to understand present difficulties in delivering care and the importance of different perspectives to stress the need for personalisation of transfusion strategy. Moreover, we learned that the process of managing more participants would have been difficult for the facilitators to manage. This number allowed everyone to have a say within a tight schedule.

## Discussion

Allogeneic stem cell transplant is the only curative option for MDS and not possible for many due to the presence of comorbidities in the predominantly elderly population. Without this curative option, patient focussed factors such as improvement, or at least maintenance, of QoL are the main aims of treatment and should be prioritised. The importance of considering what matters most to patients with MDS was emphasised during the workshop. Interestingly, the question of a cure for MDS was only raised towards the end of the workshop illustrating that patients prioritise QoL.

Using a qualitative, online workshop technique to empower patients to highlight research priority is novel and allows a greater impetus given to patients to direct resource direction to their needs. Following this pilot exercise, we plan to conduct another workshop in another part of the United Kingdom to test the extent to which different geographical and demographic groups would reach similar conclusions.

There have been few studies in MDS where QoL is the primary outcome and hence there is a paucity of evidence around best practices in relation to QoL. Empowering patients to highlight what matters most for them will help to direct further studies and resource allocation to direct patient benefit.

We have discussed some of the key outcomes from this workshop and how some of these could be addressed to improve the current outlook for people living with MDS.

### Addressing fatigue and quality of life

**Figure 2:**
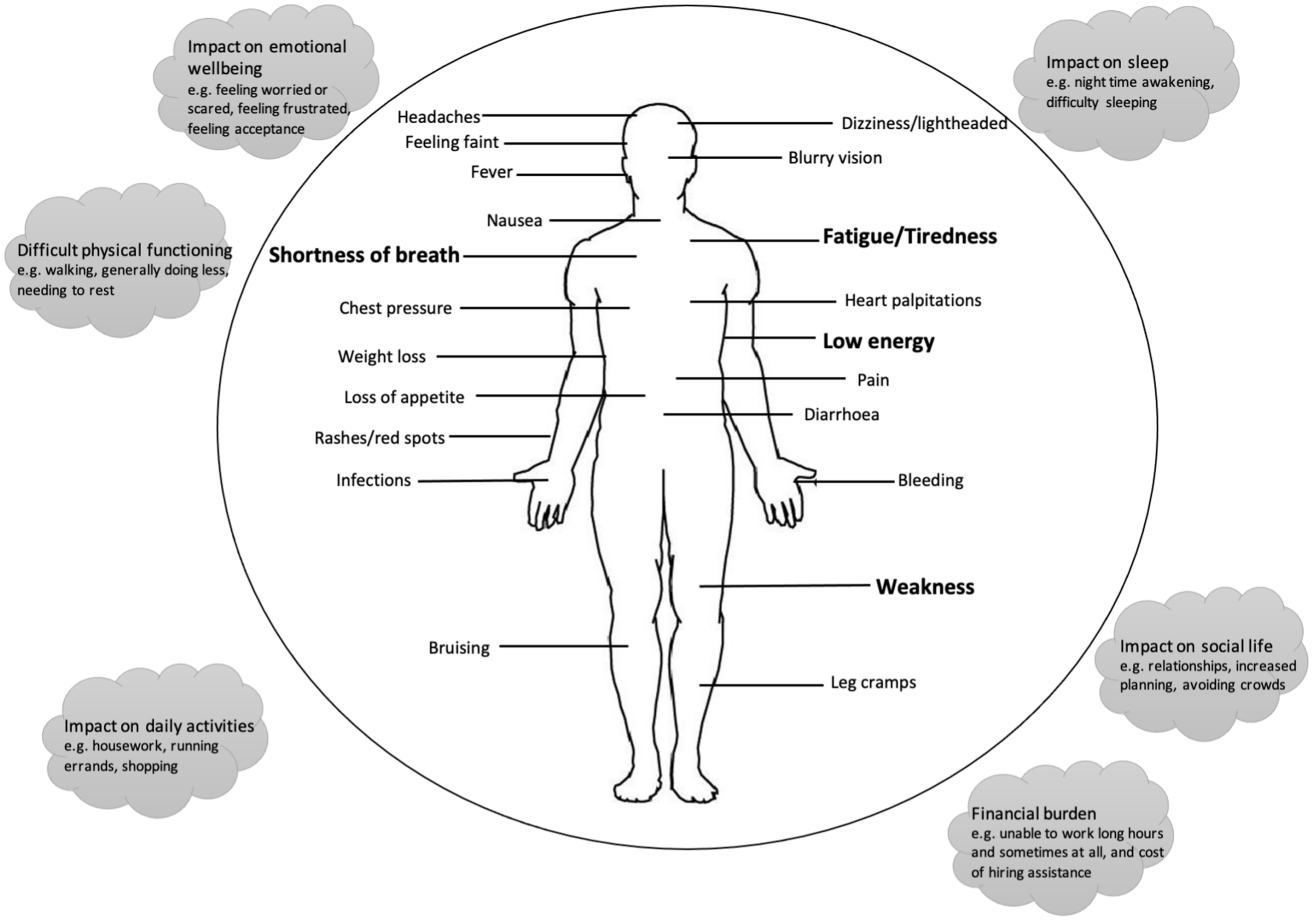
Conceptual model of patient experience with MDS (adapted from Trudeau et al. (5)).

Given the pervasiveness of anaemia in MDS, its management and the avoidance of fatigue are of vital importance. Fatigue is a complex symptom with anaemia only partly responsible for it. Other factors such as poor sleep due to pain or anxiety can be responsible. Trudeau et al. have comprehensively mapped symptoms of MDS in one cohort (5). Figure 2 displays the impacts shown in the conceptual model of patient’s experience and draws on this to highlight the holistic impacts MDS has on patients. The role of individuality in disease is complex and cannot be generally defined. In an age where individual physiological information is available, this could be used to understand the effect of various treatments on physical functions and to begin to unpick the complex relationship between other, trickier to define, symptoms such as “brain-fog,” sleep disturbance and emotional well-being.

To better address the management of fatigue, it needs to be understood as a multi-dimensional concept. Fatigue has physical and psychological components, including but not limited to; muscle weakness, lack of energy, lack of motivation and sleepiness (6). Therefore, a multi-dimensional approach, in which a variety of parameters are used to assess patient fatigue, must be implemented. Maintaining physical function is a key consideration for elderly patients. The association between Hb value and QoL (7) indicates the need to evaluate both the Hb value and QoL when deciding whether to transfuse. However, due to the prevalence of MDS in the elderly, it may be difficult to deconvolute whether QoL results from symptoms due to MDS alone or in conjunction with comorbidities or even just advancing age. Furthermore, a QoL score gives a measurement at a single time point and does not accurately represent the patient’s QoL over the course of treatment. A holistic approach would be to measure QoL scores over a treatment cycle. This would help characterise how transfusion affects fatigue in individuals over time and may help determine when is best to transfuse, and by how much.

### The role of red cell transfusion in MDS

Red blood cell transfusion is a key intervention in MDS to ameliorate the symptoms of chronic anaemia (8). Currently transfusion schedules are determined by a combination of Hb and symptoms of anaemia (9). A recurrent theme of the patient workshop was the importance of individualising use of red cell transfusions to treat symptoms of anaemia rather than to reach a “target Hb.”

Transfusion dependent (TD) patients not only suffer symptoms of anaemia but they and their carers also suffer the difficultly of the time taken, cost and travel requirements for regular transfusions and sample collection for compatibility testing. TD patients have often already been treated with erythropoietin stimulating agents with little effect and may even require iron chelating drugs with further debilitating side effects such as nausea. The decision to transfuse regularly should not be taken lightly. The benefit of each transfusion must be optimised through its timing and the quantity of blood transfused. Indeed, one study showed that 70% of patients would tolerate a temporary decrease in QoL to avoid transfusion dependence long-term (10) demonstrating the lengths some patients would go to remove the burden of transfusion dependence. Studies have shown transfusion free (TF) patients have a better QoL than TD patients (11, 12) stressing the impact this can have of patients’ lives.

Various trials regarding transfusion targets have shown mixed results. Hsia et al. (13) showed no difference in quality of life when using fresh (<7 days old) vs. standard RBC (up to 42 days old) and whilst studies show using liberal transfusion targets improve QoL (14, 15), there are conflicting results showing a prolonged increase in Hb requires more transfusions which would in turn require more time in hospital from patients. Subjective reports from patients find they can gain more prolonged benefit from larger transfusion or transfusion with a higher haematocrit (for example, from male donors). This workshop highlighted patient dissatisfaction with the “numbers-led” approach based on this single parameter. At one Hb level, variation was noted in different patients’ levels of fatigue; and within the same individual at different timepoints. This indicates Hb levels are not robust enough to determine when to transfuse, and thus manage fatigue.

There is growing evidence that treatment needs to be individualised to patients (16). The use of lifestyle monitoring devices such as smartwatches provides an opportunity to use this wealth of information to determine transfusion need.

### Predicting the disease course of MDS

Improved molecular techniques are redefining physicians’ ability to recognise high and low risk disease and relay that information to patients (17). There is much work to be done however and changes of uncertain significance are common and difficult to understand in a rare disorder. Many patients already map their disease course from diagnosis, either informally based on their symptoms or through their blood parameters and activity monitoring. A key issue of discussion in the workshops was the problem of knowing what the future holds and the need for better prognostic indicators. An example of this would be in predicting response in those who benefit to other treatments such as ESAs and luspatercept. Activity monitoring requires more formal validation but could also become a valuable tool in tracking the course of MDS and assessing patient related outcome measures. However, at present there is much uncertainty as to why some patients suffer symptoms differently to others. Some symptoms, such as mouth ulceration, are very debilitating but not life threatening and receive less attention in terms of supportive care. There is a lack of knowledge about this, and—perhaps—an unwillingness from clinicians to admit this lack of knowledge. Improving understanding and concentrating research to explain individual differences would improve patient experience.

### Current challenges in MDS landscape

This study is limited by a small sample size, time restrictions and the need to conduct the workshops online due to travel and health restrictions. However, this novel, patient led workshop technique addresses that the challenges and controversies of treating MDS are directly affecting patient satisfaction.

There are multiple challenges in the MDS landscape for patients. The enhanced diagnostic and therapeutic options make decision making more complex. This study demonstrates a novel online, interactive patient engagement method can be utilised to engage with patients where face-to-face methods are more difficult or time consuming. Patient engagement is important to improve shared decision making and research prioritisation. Pre-existing technology and future trials need to focus on predicting disease course and maximising the benefits of interventions of supportive care.

## Data availability statement

The raw data supporting the conclusions of this article will be made available by the authors, without undue reservation.

## Ethics statement

Ethical approval was not required for the studies involving humans because ethical approval not required for to run an involvement activity as per Oxford University RGEA classification group and NIHR (https://www.spcr.nihr.ac.uk/PPI/resources-for-researchers/faq/do-i-need-ethical-approval-to-run-an-involvement-activity). The studies were conducted in accordance with the local legislation and institutional requirements. The participants provided their written informed consent to participate in this study.

## Author contributions

MP: Writing – original draft, Writing – review & editing. CG-H: Writing – original draft, Writing – review & editing. ID-H: Writing – review & editing. EC: Writing – review & editing. SS: Writing – review & editing. MM: Writing – review & editing. NR: Writing – review & editing.
